# Preliminary evidence from microsatellite and 2La inversion polymorphism suggests DDT resistance drives genetic differentiation between populations of *Anopheles gambiae s.s.* in South West Nigeria

**DOI:** 10.1186/1475-2875-13-S1-P1

**Published:** 2014-09-22

**Authors:** Adedapo O Adeogun, Kehinde OK Popoola, Lizette L Koekemoer, Basil D Brooke, Adedayo O Oduola5, Samson T Awolola

**Affiliations:** 1 Molecular Entomology and Vector Control Research Laboratory, Nigerian Institute of Medical Research, PMB 2013, Yaba, Lagos, Nigeria; 2 Department of Zoology, University of Ibadan, Ibadan, Nigeria; 3 Center for Opportunistic, Tropical & Hospital Infections, National Institute for Communicable Diseases, Johannesburg, South Africa; 4 Wits Research Institute for Malaria, School of Pathology, Faculty of Health Sciences, University of the Witwatersrand, Johannesburg, South Africa; 5 Department of Zoology, University of Ilorin, Kwara State, Nigeria

## Background

Resistance to the insecticide DDT has emerged in several *Anopheles gambiae* populations which has stalled its use in malaria vector control programs in Nigeria. Data on insecticide resistance in Nigeria are still patchy with consequent lack of understanding of resistance mechanisms. This study was therefore designed to investigate the relationship between DDT resistance, inversion 2La and microsatellite loci in *Anopheles gambiae s.s.* populations from Lagos and Oyo State, South West, Nigeria.

## Materials and methods

*Anopheles* larvae were collected from six localities each in Lagos and Oyo State. These were reared to adults and 3-5 day old adults were exposed to 4% DDT according to the standard WHO protocol. Resistance profiles were compared between Lagos and Oyo State samples using a standard *t-test*. All *Anopheles* mosquitoes exposed were identified to species using external morphology followed by PCR assay and restriction enzyme digest. Underlying genetic differentiation was first determined by conducting 2La inversion PCR on 231 *Anopheles gambiae s.s.* DDT resistant samples selected randomly from all localities. Inversion karyotype data was compared between groups (Lagos and Oyo State) using standard F_ST_ estimates. Genetic differentiation was further scrutinized by assessing the assortment of 10 microsatellites by PCR using the DNA products from the inversion analysis. Microsatellite data were analyzed with one locus estimates following standard ANOVA.

## Results

*Anopheles* mosquitoes were resistant to DDT in all the localities surveyed with significant variation in mortality (at *P* < 0.05) between the two groups. All *Anopheles* from Lagos and Oyo State used for the study were morphologically identified as *Anopheles gambiae s.l.* PCR assay identified all Lagos samples as *An. gambiae s.s.* and *An. coluzzii* while *An. gambiae s.s.* and *An. arabiensis* constitute the Oyo State population with sympatric occurrence of *An. coluzzii* and *An. gambiae* The resistance profile showed the same trend with chromosomal inversion 2La profile. The F_ST_ for inversion 2La was 0.09 and 0.07 for Lagos and Oyo State respectively (*P* > 0.05). Microsatellite locus estimate revealed that four loci (AG2H26, AG2H637, AG2H772 and AG2H603) located within and outside inversion 2La accounted for the bulk of genetic differentiation between populations.

## Conclusions

These data suggest that inversion 2La and four microsatellite loci are associated with the development of resistance to DDT in *Anopheles gambiae s.s.* populations from Lagos and Oyo States in South West Nigeria. Microsatellite analysis shows that gene flow is extensive across all loci and that assortment of the four loci located within or close to inversion 2La suggests that the primary factor controlling DDT resistance is likely within or close to inversion 2La*.* A candidate gene that may at least partially account for these data is the *kdr* locus which is located near the centromere of arm 2L.

